# Targeting the mTOR pathway uncouples the efficacy and toxicity of PD-1 blockade in renal transplantation

**DOI:** 10.1038/s41467-019-12628-1

**Published:** 2019-10-17

**Authors:** Khashayar Esfahani, Tho-Alfakar Al-Aubodah, Pamela Thebault, Réjean Lapointe, Marie Hudson, Nathalie A. Johnson, Dana Baran, Najwa Bhulaiga, Tomoko Takano, Jean-François Cailhier, Ciriaco A. Piccirillo, Wilson H. Miller

**Affiliations:** 1 Departments of Medicine and Oncology, Segal Cancer Center, Rossy Cancer Network, McGill University, Montréal, Québec Canada; 2 Centre of Excellence in Translational Immunology (CETI), Montréal, Québec Canada; 3 McGill Center for Translational Research in Cancer, McGill University, Montréal, Québec Canada; 4 Department of Microbiology and Immunology, McGill University, Montréal, Québec Canada; 5 Program in Infectious Diseases and Immunity in Global Health, Centre for Translational Biology, Research Institute of the McGill University Health Centre, Montréal, Québec Canada; 6 University of Montréal Hospital Research Centre, Montréal, Québec Canada; 7 Institut du cancer de Montréal, Montréal, Québec Canada; 8 Clinical Immuno-Monitoring Core Facility, CRCHUM, Montréal, Québec Canada; 9 Department of Medicine, University of Montréal, Montréal, Québec Canada; 10 Department of Medicine, Division of Rheumatology, Lady Davis Institute, Jewish General Hospital, McGill University, Montréal, Québec Canada; 11 Department of Medicine, Division of Hematology, Lady Davis Institute, Jewish General Hospital, McGill University, Montréal, Québec Canada; 12 Department of Medicine, Division of Nephrology, Faculty of Medicine, McGill University, Montréal, Québec Canada

**Keywords:** Nephrology, Cancer therapy

## Abstract

Immune checkpoint inhibitor (ICI) use remains a challenge in patients with solid organ allografts as most would undergo rejection. In a melanoma patient in whom programmed-death 1 (PD-1) blockade resulted in organ rejection and colitis, the addition of the mTOR inhibitor sirolimus resulted in ongoing anti-tumor efficacy while promoting allograft tolerance. Strong granzyme B^+^, interferon (IFN)-γ^+^ CD8^+^ cytotoxic T cell and circulating regulatory T (T_reg_) cell responses were noted during allograft rejection, along with significant eosinophilia and elevated serum IL-5 and eotaxin levels. Co-treatment with sirolimus abated cytotoxic T cell numbers and eosinophilia, while elevated T_reg_ cell numbers in the peripheral blood were maintained. Interestingly, numbers of IFN-γ^+^ CD4^+^ T cells and serum IFN-γ levels increased with the addition of sirolimus treatment likely promoting ongoing anti-PD-1 efficacy. Thus, our results indicate that sirolimus has the potential to uncouple anti-PD-1 therapy toxicity and efficacy.

## Abstract (web summary)

The use of immune checkpoint inhibitors (ICI) in cancer patients with solid organ allografts is hampered due to potential organ rejection. Here, the authors present a case report of a patient with kidney allograft and show that treatment with the mTOR inhibitor sirolimus preserves peripheral tolerance and anti-tumour efficacy of ICI therapy.

## Introduction

Immune checkpoint inhibitors (ICIs) are among the most promising approaches to fighting cancer^1^. Immune checkpoints, such as the programmed-death 1 (PD-1) or the cytotoxic T-lymphocyte-associated protein 4 (CTLA-4), are physiological immunoinhibitory and regulatory components of the immune system that counteract immune activation to maintain immune homeostasis, promote self-tolerance, and protect against autoimmunity. ICIs interfere with these immunoinhibitory signals, thus promoting immune activation, and enhancing naturally occurring anti-tumor immunity. The lack of specificity of ICIs is, on the other hand, responsible for undesirable off-target immune and inflammatory events known as immune-related adverse events (irAEs)^2^. Thus, it is important to uncouple the anti-tumorigenic effects of ICIs from its capacity to induce irAEs.

Upon antigen recognition and cellular activation, naïve T cells undergo rapid expansion, and differentiation into distinct functional subsets. The energy requirements for this expansion is significant, and T cell activation is accompanied by dramatic changes in cellular metabolism. Indeed, these metabolic changes orchestrate significant control on T cell differentiation, serving as a strong determinant between inflammatory and immunoregulatory T cell functions^3–5^. Hence, while PD-1 inhibitors lead to widespread T cell activation, the mammalian target of rapamycin (mTOR), a central integrator of the metabolic and immune signals associated with T cell activation, is emerging as a critical driver and modulator of T cell differentiation, function, and homeostasis^6^.

A particularly difficult therapeutic challenge is presented by patients with renal transplants who are candidates for ICIs. Cancer immunotherapy results in organ rejection in the majority of these patients, requiring more immunosuppression to prevent allograft rejection and graft loss^7–9^. This, of course, counteracts the clinical benefits of ICIs^9,10^. While the mechanisms of classic allograft rejection have been extensively studied, the immune mechanisms leading to allograft rejection while on ICIs remain poorly understood^11^. For patients with kidney transplants, anecdotal use of mTOR inhibition (mTORi) as primary prevention was reported^12,13^. However, not all patients with a renal allograft will experience rejection, and no data exists on the use of mTORi for secondary prevention^14^.

Here, we present a detailed immunophenotyping of peripheral blood from a patient who underwent kidney allograft rejection and some irAEs while on PD-1 blockade and was subsequently controlled using ICI-mTORi combination therapy. Allograft rejection featured significant activation and proliferation of peripheral CD4^+^ and CD8^+^ T cells, heavy eosinophilia, and elevated serum levels of pro-inflammatory cytokines and chemokines. There was successful control of inflammation with steroid treatment followed by ICI-mTORi combination. Nevertheless, ICI-mTORi combination therapy maintained elevated frequencies of CD4^+^ and CD8^+^ T cells producing the pro-inflammatory cytokine interferon (IFN)-γ in peripheral blood, as well as increased numbers of regulatory T (T_REG_) cells, an adaptive immune subset critical for allograft tolerance and tissue homeostasis. Thus, our data indicate that the inclusion of mTORi to PD-1 inhibition could change the immune landscape in favor of allograft preservation without compromising anti-tumor efficacy.

## Results

Our patient is a 57-year old female who received an HLA identical kidney from her sister in 1985. She had end stage renal disease from membranoproliferative glomerulonephritis (GN). Following the transplant, she was on triple therapy with tacrolimus, mycophenolate mofetil, and low dose daily prednisone. Allograft function was excellent, with no episodes of rejection or evidence of GN recurrence for 32 years.

In 2016, she was diagnosed with a T4aN2a primary melanoma of the right upper arm, which was treated with surgery and axillary dissection. She did not receive any adjuvant therapy and was kept on baseline immunosuppression, the benefits of allograft tolerance outweighing the potential negative association of immunosuppression in the setting of de novo skin malignancy^15^. A year later, she was diagnosed with a large axillary recurrence of her melanoma (Supplementary Fig. S1A). The recurrence was not amenable to surgical resection or radiation, given the proximity of the tumor to key vessels and the brachial plexus, as well as the high risk for lymphedema. Therefore, treatment with a PD-1 inhibitor (pembrolizumab) was given. All three immunosuppressive medications were stopped two weeks prior to ICI initiation to maximize anti-tumor efficacy, and this timepoint (Week 0 [W0]) marks the beginning of our data and biological specimen collections. There was no sign of renal allograft rejection up to W2, when pembrolizumab was initiated. On W7, the patient’s axillary mass had decreased from total conglomerate dimensions of 13.8 × 6.4 cm to 7.5 × 3.6 cm on CT scans, thus representing a partial response according to RECIST 1.1 criteria (Supplementary Fig. 1A). However, on W9, she developed an acute rise in serum creatinine from 57 to 310 μmol/L (reference range 55–110 μmol/L), as well as other irAEs, consisting of a grade 2 diffuse body maculopapular rash and a grade 2 colitis (as per Common Terminology Criteria for Adverse Events [CTCAE]). The patient was hospitalized and started on solumedrol 500 mg IV per day for three days, followed by a transition to oral prednisone at 1 mg/kg. All irAEs subsided, and creatinine returned to normal by W11. Once prednisone was fully tapered, sirolimus at 2.5 mg PO daily was added on W15, and PD-1 therapy was resumed a week later (W16). The patient did not experience any further irAEs or allograft rejection and had ongoing anti-tumor benefit, as demonstrated by a 3.6 cm short axis diameter of the axillary mass, representing stable disease per RECIST 1.1 (Supplementary Fig. 1A), at a 6-month follow-up scan on W22. Sirolimus dosage was monitored and adjusted as necessary to maintain therapeutic plasma levels (5–9 ng/mL).

### ICI-mTORi combination therapy dampens activation and proliferation of T cells post irAEs

**Fig. 1:**
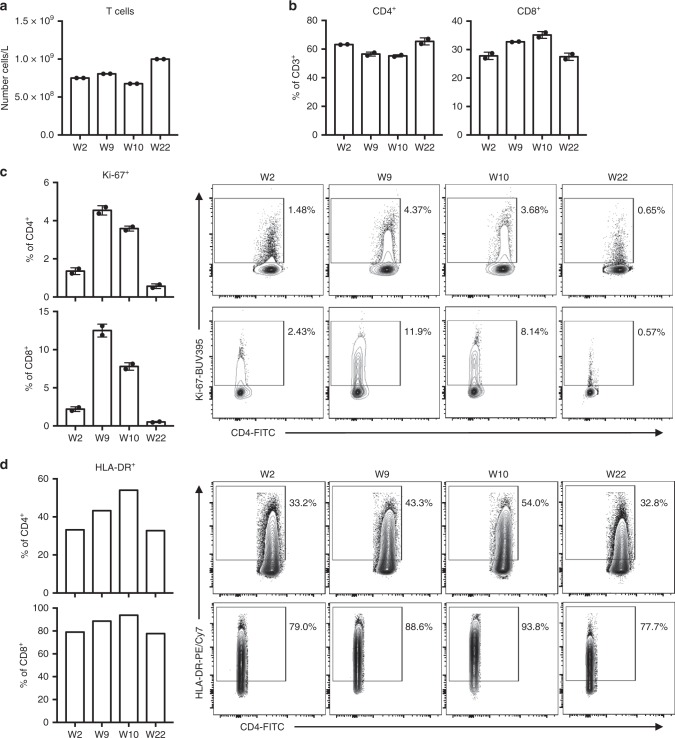
T cell activation during allograft loss is ameliorated with ICI-mTORi combination. Peripheral blood mononuclear cells (PBMC) were isolated from the melanoma patient before pembrolizumab therapy and renal transplant rejection (W2), during renal transplant rejection when following two cycles of pembrolizumab therapy (W9), at the height of renal transplant rejection when prednisone therapy was started and pembrolizumab halted (W10), and finally when rejection was resolved, prednisone tapered away and the patient was placed on pembrolizumab and sirolimus combination therapy (W22). PBMC were stained for flow cytometric analysis of various T cells populations. **a** Total T (CD3^+^) cell numbers in PBMC as determined by flow cytometry are plotted. **b** Proportions of CD4^+^ and CD8^+^ T cells are shown. **c**, **d** Proportions of cycling (Ki-67^+^) and activated (HLA-DR^+^) cells of CD4^+^ or CD8^+^ T cells are shown with the representative flow cytometry plots on the right. **a**–**c** were completed in duplicates, mean ± SD are shown. **d** was completed once

Since PD-1 and mTORi are known to alter T cell responses in vivo, we carried out in-depth, multi-parametric flow cytometric analyses of various T cell populations in the peripheral blood of our patient (Supplementary Figs. 2–4). Total circulating T cell (CD3^+^) numbers did not change substantially during renal rejection (W9 and W10) in comparison to pre-anti-PD-1 exposure (W2) (Fig. 1a). When further stratifying T cells into CD4^+^ and CD8^+^ subsets, the latter being notably involved in renal allograft rejection, we noticed an increase in the proportions of circulating CD8^+^ T cells over CD4^+^ T cells at the time of rejection, a trend which was reversed under ICI-mTORi (Fig. 1b). To determine whether CD4^+^ and CD8^+^ T cells were activated during rejection, we assessed cellular expression of HLA-DR, a T cell activation marker, and Ki-67, a cell cycle marker expressed by non-quiescent cells. The frequency of activated (HLA-DR^+^) and cycling (Ki-67^+^) CD4^+^ and CD8^+^ T cells increased greatly during rejection but returned to baseline levels under ICI-mTORi, indicating a role for sirolimus in hindering T cell activation and thereby preventing the expansion and accumulation of CD8^+^ T cells (Fig. 1c, d).

We subsequently sought to validate the potential of mTORi to promote T cell quiescence in vitro. To this end, we activated T cells in patient or HC PBMC with anti-CD3 in the presence or absence of sirolimus. Four days into the culture, proportions of cycling (Ki-67^+^) CD4^+^ and CD8^+^ T cells were significantly lower in both patient and the HC PBMC under sirolimus treatment than DMSO control (Supplementary Fig. 5A). Nevertheless, T cells were still activated in the presence of sirolimus since CD25 expression here was similar to the DMSO control (Supplementary Fig. 5B). This indicates that while T cells were efficiently activated, a large proportion failed to proliferate and partake in any downstream immune response. Taken together, the dampened CD4^+^ and CD8^+^ T cell proliferation observed ex vivo following restoration of ICI was likely the result of mTORi co-therapy.

### Rejection is marked by significant IL-5 upregulation, whereas return to normal renal function and immune surveillance correlated with strong IFN-γ upregulation

**Fig. 2:**
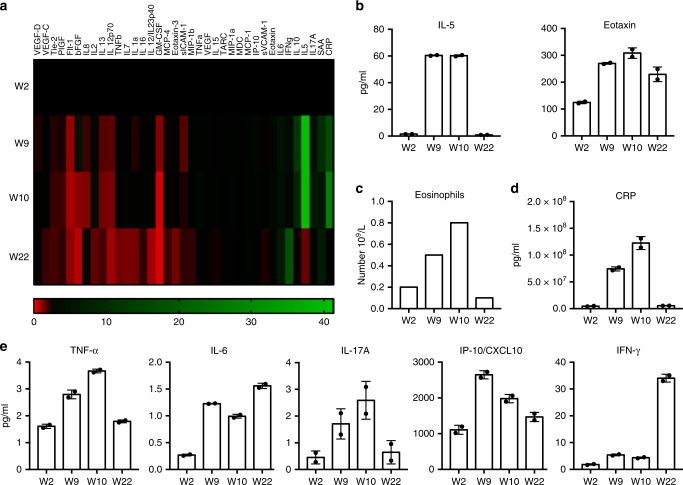
Analysis of inflammatory mediators during ICI-mTORi combination therapy. **a** Cytokine and chemokine expression levels were determined using a multiplex cytokine assay. Each column represents a specific cytokine or chemokine as indicated at the top of the heat map. The four time points are defined on the left side of the figure and the relative levels of each circulating cytokine, compared to baseline, is color-coded according to the gauge at the bottom of the figure. Absolute values of **b** interleukin (IL)-5, eotaxin, **d** CRP **e** TNF-α, IL-6, IL-17A, IP-10/CXCL10, and interferon (IFN)-γ are shown at the four treatment time points. All cytokine measurements were completed in duplicates, mean ± SD are shown. **c** Absolute numbers of eosinophils as were determined by complete blood cell counts from blood taken at the four different time points are reported

Given the significant changes in the dynamics of T cell responses observed during rejection and quiescent phases, we subsequently sought to map the inflammatory landscape by measuring 37 cytokines/chemokines circulating in the plasma at the same four time points for which immunophenotyping was performed (Fig. 2a). During rejection, we observed an upregulation of cytokines and chemokines associated with eosinophils (IL-5 and eotaxin) (Fig. 2b), which correlated with a concomitant, strong increase in the numbers of circulating eosinophils and another pro-inflammatory mediator, C-Reactive Protein (CRP) (Fig. 2c, d). Modest increases in other inflammatory cytokines, including tumor necrosis factor-alpha (TNF-α), interleukin (IL)-6, and IL-17A were observed (Fig. 2e). Similarly, the pro-inflammatory chemokine IP-10/CXCL10, a molecule implicated in renal allograft loss, was very significantly elevated at this point (Fig. 2e)^16,17^. During anti-PD-1-mTORi combination therapy, eosinophilia and the levels of all the above inflammatory mediators, except IL-6 and eotaxin, returned to baseline (Fig. 2b-d). Strikingly, serum levels of (IFN)-γ, an important anti-tumor cytokine, were significantly elevated at this point (Fig. 2e). Overall, these results suggest that while irAEs-related inflammation was abated systemically with the addition of corticosteroids and mTORi, the increased IFN-γ may indicate continued and efficient tumor control.

### Elevated levels of circulating IFN-γ-producing T cells are maintained on ICI-mTORi combination therapy

**Fig. 3:**
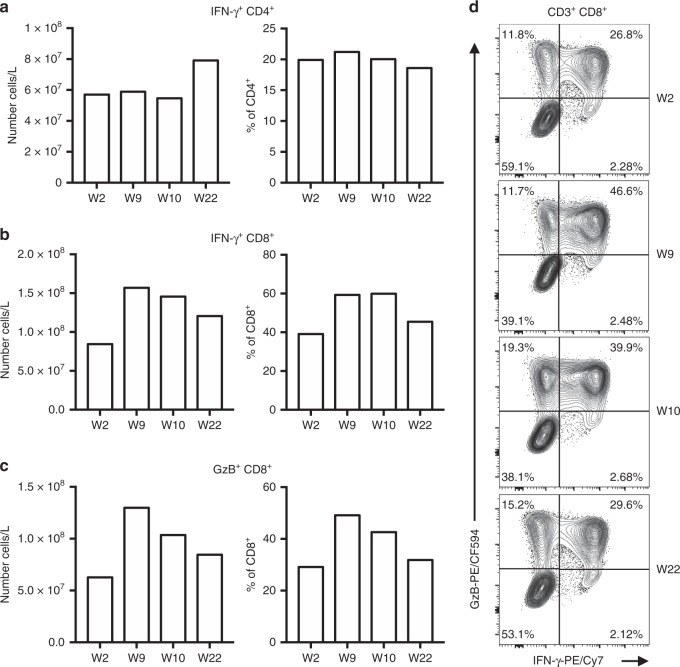
Peripheral IFN-γ-producing T cells remain elevated under ICI-mTORi combination therapy. PBMC were stimulated with phorbol 12-myristate 13-acetate (PMA) and ionomycin and treated with GolgiStop (monensin solution) for 4 h before intracellular flow cytometric staining of various cytokines. **a**, **b** The number and proportions of IFN-γ^+^ (**a**) CD4^+^ and (**b**) CD8^+^ T cells are shown. **c** The number and concentration of granzyme B (GzB)-producing CD8^+^ T cells in peripheral blood are shown. **d** Sample flow cytometry plots of CD8^+^ T cell cytokine production are provided. Flow cytometric analyses were completed once

Given the important role of T cell-derived IFN-γ in anti-tumor immune surveillance, we sought to determine whether the frequency of IFN-γ producing T cells was impacted by mTORi. To this end, we polyclonally stimulated PBMC from each treatment group, stained for cytokines intracellularly, and assessed the frequency of cytokine-producing cells by flow cytometry. Numbers of IFN-γ^+^ CD8^+^ cells, but not IFN-γ^+^ CD4^+^ T cells, were elevated during allograft rejection when compared to baseline levels (Fig. 3a, b). Moreover, these IFN-γ^+^ CD8^+^ T cells largely expressed granzyme B, a secreted serine protease that mediates CD8^+^ T cell cytotoxic functions (Fig. 3c). Interestingly, the addition of mTORi for irAE control resulted in increased numbers of CD4^+^ T cells producing IFN-γ, although their proportions remained unchanged (Fig. 3a). Among CD8^+^ T cells, the fraction of IFN-γ-producing cells decreased under ICI-mTORi but remained higher than baseline levels (Fig. 3b). Thus, pro-inflammatory CD4^+^ and CD8^+^ T cells remained in circulation during the immune quiescent phase.

A similar trend was observed with in vitro treatment of PBMC with sirolimus. Indeed, while sirolimus decreased the proportions of IFN-γ-producing CD4^+^ T cells, it did not affect the proportions of IFN-γ-producing CD8^+^ T cells (Supplementary Fig. 5C, D). Overall, these results suggest that while the addition of mTORi was crucial in controlling the activation and expansion of cytotoxic CD8^+^ T cells, as shown earlier, IFN-γ^+^ CD8^+^ T cells with potential anti-tumor functions were still found in greater numbers in peripheral blood when compared to baseline.

### Elevated T_reg_ cell numbers in circulation are maintained following ICI-mTORi

**Fig. 4:**
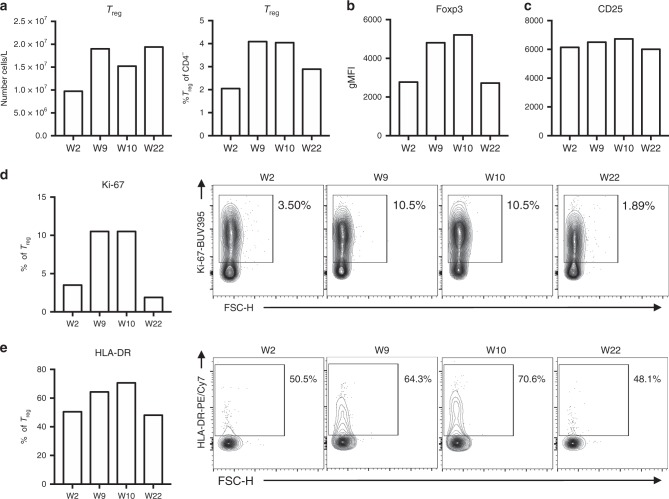
Increased T_reg_ cell numbers were maintained in peripheral blood under ICI-mTORi therapy (**a**) T_reg_ cell (CD3^+^CD4^+^CD25^High^CD127^Low^Foxp3^+^Helios^+^) numbers and proportions from total CD4^+^ T cells in blood are plotted as was determined with flow cytometry. (**b**, **c**) Geometric mean fluorescence intensity (gMFI) of (**b**) Foxp3 and (C) CD25 on T_reg_ cells are shown. (**d**, **e**) Proportions of (**d**) cycling (Ki-67^+^) and (**e**) activated (HLA-DR^+^) cells of T_reg_ cells are shown with the representative flow cytometry plots on the right. Flow cytometric analyses were completed once

Finally, given the central role FOXP3^+^ T_reg_ cells play in maintaining immune tolerance within both the allograft and the tumor, we sought to determine how T_reg_ cellular dynamics were changed under ICI-mTORi combination treatment. Stably suppressive T_reg_ cell (CD3^+^ CD4^+^ CD25^High^ CD127^Low^ Foxp3^+^ Helios^+^) numbers and proportions increased substantially during the rejection phase and remained elevated following ICI-mTORi (Fig. 4a). During the rejection phase, expression of FOXP3, the master lineage-defining transcription factor of T_reg_ cells, increased substantially but was restored to baseline levels with mTORi and PD-1 blockade (Fig. 4b). We further observed that T_reg_ cells at the peak of rejection expressed only slightly higher levels of CD25 which returned to normal levels following ICI-mTORi co-treatment (Fig. 4c). As with other T cell subsets, T_reg_ cells demonstrated a non-quiescent (Ki-67^+^) and activated (HLA-DR^+^) phenotype during the rejection phase that was suppressed by ICI-mTORi therapy (Fig. 4d, e). Altogether these data indicate that T_reg_ cell activation and expansion increased in response to allograft rejection, and that although T_reg_ cell proliferation and activation was dampened by ICI-mTORi co-therapy, T_reg_ cell numbers remained elevated thereafter.

## Discussion

The successful activation of the immune system under ICI therapy leads to a myriad of functional changes and expansion of various immune cell subsets, and secretion of a complex network of immunomodulatory cytokines. Although strong immune activation is the desired outcome to enhance naturally occurring anti-tumor immune surveillance, there is also a significant risk for autoimmunity. The mTOR pathway has long been recognized to play a pivotal role in integrating signals at the cellular level and promote autoimmunity and carcinogenesis^18,19^. Conversely, mTOR inhibitors, such as sirolimus, are frequently used to maintain immune tolerance, prevent allograft rejection, and are occasionally used as anti-neoplastic treatment. Although the mTOR pathway can be activated in the majority of melanomas, mTOR inhibitors have not been proven effective in this clinical setting^20–22^. However, under immune checkpoint inhibition, where the risk of allograft rejection for solid organ transplants is high, we hypothesized that mTORi might support immune tolerance, while potentially adding anti-tumor efficacy to PD-1 blockade for patients with metastatic melanoma. In addition, we sought to investigate the effects of the ICI-mTORi combination on the immune landscape, as this has not been previously reported.

In this case report, we demonstrated that ICI-induced kidney allograft rejection was associated with cytotoxic CD8^+^ T cell activation in the periphery, a subset of cells with a well-established role in renal allograft rejection on anti-PD-1 therapy^23,24^. However, during a re-challenge with pembrolizumab in combination with sirolimus, global T cell activation and proliferation was abated, although IFN-γ-producing CD4^+^ T cells and cytotoxic CD8^+^ T cells persisted in circulation. Thus, ICI-mTORi combination therapy promoted a state of functional tolerance without a loss of immune-mediated anti-tumor activity. Indeed, both our in vivo and in vitro results suggest that sirolimus prevented adverse T cell responses in this one patient.

During allograft rejection, our cytokine analysis showed a very strong induction of IL-5, a cytokine that plays a crucial role in eosinophil activation and accumulation. This was also accompanied by the upregulation of the eosinophil-recruiting chemokine, eotaxin, and significant peripheral blood eosinophilia. Although a biopsy was not performed in our patient, eosinophil infiltration is a prominent marker of allograft rejection and predicts a poor prognosis^25,26^. Recent reports suggest that eosinophilia is a toxicity frequently associated with anti-PD-1 therapy^27^. With the addition of mTORi, eosinophil levels returned to the normal range upon re-challenge with the anti-PD-1. The effect of sirolimus on the eosinophils remains poorly understood, but animal models suggest that rapamycin may prevent terminal differentiation of eosinophils^28^. IP-10/CXCL10 was also significantly increased during renal allograft rejection and returned to baseline levels following re-challenge with anti-PD-1 under mTORi. This pro-inflammatory chemokine is a well-established urinary, serum and tissue biomarker of renal allograft loss. In this patient, several other inflammatory cytokines, mainly IL-6, TNF-α and IL-17A, were also elevated during the rejection phase, and were likely promoting the observed concomitant skin and gastrointestinal toxicities^29–31^. The levels of most of these cytokines returned to baseline following high dose steroid administration and did not rise upon rechallenge with ICI-mTORi. Our patient also did not have any recurrent skin or gastrointestinal toxicities.

Another striking feature of our analysis was the strong upregulation of circulating IFN-γ levels under ICI-mTORi combination therapy. IFN-γ is a well-established pleiotropic cytokine with the power of exerting opposite allograft outcomes depending on the immune landscape. Although generally a pathogenic factor for renal allograft rejection, IFN-γ has been shown to promote allograft tolerance in certain settings by counteracting the effects of both IL-5 and IL-6, which are strong mediators of allograft rejection, as previously published and also observed in our case^26,32–36^. In regards to tumor control, IFN-γ has pleiotropic effects, and is critical for anti-PD-1 efficacy by promoting immune surveillance^37^. Although the IFN-γ and the mTOR pathways seem to be tightly interwoven, no data exist on the in vivo outcomes of mTORi on IFN-γ signaling and function^38–40^. In our case, the addition of sirolimus did not seem to impair the numbers of IFN-γ secreting T cells and the high circulating levels of this cytokine.

The outcome of T cell activation is guided by multiple cues derived from the T cell microenvironment. mTOR is a central integrator of these signals, playing a critical role in driving T cell differentiation and function and controlling multiple metabolic programs. Interestingly, mTOR inhibition has been described to promote the induction and maintenance of FOXP3 expression and consequential T_reg_ cell development in humans^41^. We also sought to examine the dynamics of T_reg_ cells, before and after combination therapy with PD-1 inhibitor and mTORi. A strong activation and expansion of T_reg_ cells, with higher expression of Foxp3, was evident during the rejection episode, suggesting the engagement of strong immunosuppression in response to inflammation^42^. A similar compensatory delayed expansion of T_reg_ cells in response to inflammation is known to control immune responses and maintain peripheral tolerance in various disease systems^43^. Interestingly, T_reg_ cell numbers were maintained during ICI-mTORi combination therapy suggesting ongoing allograft protection. While Foxp3 expression was restored to baseline with mTORi-ICI combination, CD25 expression on T_reg_ cells was only slightly modulated, indicating that T_REG_ cells in the periphery maintained their robust regulatory phenotype throughout the clinical trajectory of the patient^44^. In accordance with T_reg_ cell expansion during allograft rejection, there was a substantial increase in serum IL-10—a potent immunomodulatory cytokine produced by protective immune cell subsets, including T_reg_ cells, and known to play a key role in inducing and maintaining allograft tolerance^45^. Thus, our results suggest that the selective use of mTORi in combination with PD-1 blockade may promote and/or maintain T_reg_ cell functions amidst evolving anti-tumor immune responses and prevent graft rejection.

Our work has a few limitations. A tumor and/or renal biopsy during the immune-related adverse events would have been helpful to assess immune crosstalk between the site of toxicity and the tumor microenvironment but could not be performed in timely manner due to the therapeutic anticoagulation reversal (for the patient’s upper extremity thrombosis) that was required. Furthermore, albeit allograft tolerance would have been unlikely upon a rechallenge with a PD-1 alone, it is possible that the patient would not have experienced further extra-renal immune toxicities. Following the resolution of the toxicities, the patient preferred to continue treatment to maximize tumor response, all at the same time accepting the risk of dialysis and renal rejection, when an alternative would have been to adopt a watch and wait strategy.

In summary, immune profiling of one exceptional patient provides supporting data for the combination of mTORi in combination with ICIs to prevent graft rejection without compromising anti-tumor efficacy. Key recent reviews of all the transplant cases treated with ICIs highlight the vast heterogeneity of the immunosuppression regimens used in this setting the void of any translational data guiding treatment decisions^7–9^. Given the severe and potentially life-threating complications associated with allograft rejection, our data are timely in providing strong basic science, as well as clinical rationale for the ICI-mTORi combination as a potential standard of care for these patients. Clinical trials investigating similar immunomodulatory approaches are underway (ClinicalTrials.gov Identifier: NCT03816332). Our patient, who also developed skin and gut irAEs, did not have a recurrence of these immune toxicities under ICI-mTORi, raising the hypothesis that mTORi played a role in abating further immune toxicities. As such, its use warrants further investigation beyond the setting of renal transplantation.

## Methods

### Sample collection and processing

The patient and a healthy control consented to have peripheral blood collection for this research in accordance to the Research Ethics Board approved protocol CODIM-MBM-17–041 at the Jewish General Hospital, in Montréal, Canada. Similarly, both the patient and the healthy control provided written informed consent for the publication of these results. We performed peripheral blood immunophenotyping and analysis of circulating cytokine levels at four different time points (W0 marking the stop of all immunosuppression):

1. Week 2: Pre-PD-1 blood was collected before initiation of PD-1.
2. Week 9: Onset of rejection was marked by the first documented rise in creatinine levels, at which time the patient also manifested skin and gut toxicities.
3. Week 10: Peak of rejection was defined as the time when creatinine was at its highest, before any improvement. At this moment, the patient had received three days of high dose IV solumedrol. Following this timepoint collection, the patient continued on a transition to oral prednisone 1 mg/kg and a taper over 4 weeks.
4. Week 22: The last time point was 3 months after resolution of the allograft rejection, while the patient was on pembrolizumab in combination with sirolimus. At this time, tumor response was evident on imaging and the patient did not have any toxicities from ICI.

Peripheral blood collected in EDTA coated tubes at the above time points was centrifuged at 1500 × *g* for 20 min. The top plasma layer was aliquoted into cryovials and frozen at −80 °C. The cellular layer was diluted in phosphate-buffered saline (PBS) and was gently layered onto Ficoll medium before centrifuging with no brake at 800 × *g* for 20 min. The opaque interface containing the peripheral blood mononuclear cells (PBMC) were collected and frozen in 10% dimethyl sulfoxide (DMSO) and 90% fetal bovine serum (FBS) medium at −80 °C overnight before transfer to liquid nitrogen for long-term storage. The patient consented to publication of this research.

### Multi-parametric flow cytometry

For ex vivo immunophenotyping, PBMC were thawed and rested for 2 h at 37 °C. Half of the PBMC were stimulated using phorbol 12-myristate 13-acetate (PMA) (25 ng/ml) and ionomycin (1 μg/ml) (Sigma-Aldrich) in the presence of GolgiStop (BD Biosciences, catalog: 554724, 1:1000) for 4 h for flow cytometric cytokine analysis. For in vitro cultures, cells were stained directly after 96 h of cell culture. Cells were stained with Fixable Viability Dye eFluor 780 (Invitrogen, 65-0865-18, 1:1000). Extracellular staining was carried out using antibodies raised against human cell surface antigens: anti-CD3ε BV785 (OKT3, Biolegend, 317330, 1:100), anti-CD4 FITC (RPA-T4, BD Biosciences, 561842, 1:50), anti-CD8α V500 (RPA-T8, BD Biosciences, 560774, 1:100), anti-CD25 APC (M-A251, BD Biosciences, 555434, 1:5), anti-CD25 BV605 (2A3, BD Biosciences, 562660, 1:100), anti-CD127 PE-eFluor 610 (eBioRDR5, Invitrogen, 61-1278-42, 1:40), anti-CD45RA Alexa Fluor 700 (HI100, BD Biosciences, 560673, 1:40), anti-TIGIT PerCp-eFluor 710 (MBSA43, Invitrogen, 46-9500-42, 1:20), anti-CD307c biotinylated (H5, BD Biosciences, 565056, 1:20), anti-HLA-DR biotinylated (LN3, Invitrogen, 13-9956-82, 1:40), and streptavidin conjugated to PE/Cy7 (Invitrogen, 25-4317-82, 1:200). Cells were fixed and permeabilized using a Foxp3 Transcription Factor Fixation/Permeabilization buffer set (eBioscience). Intracellular staining was carried out in permeabilization buffer using antibodies raised against human intracellular proteins: anti-Foxp3 PE (236A/E7, Invitrogen, 12-4777-42, 1:20), anti-Helios Pacific Blue (22F6, Biolegend, 137220, 1:20), anti-Ki-67 BUV395 (B56, BD Biosciences, 564071, 1:50), anti-IL-2 PerCP/Cy5.5 (MQ1-17H12, BD Biosciences, 560708, 1:20), anti-IFN-γ PE/Cy7 (4S.B3, BD Biosciences, 557844, 1:100), and anti-Granzyme B PE-CF594 (GB11, BD Biosciences, 562462, 1:50). PBMC stimulated with PMA and ionomycin for cytokine production were stained intracellularly for CD4 and CD8α expression. Cells were acquired using the BD LSRFortessa X-20 and analyzed on the FlowJo v.10 analysis platform (FlowJo, LLC).

### Cell culture

PBMC from week 22 (see under “Sample collection and processing”) and obtained from a 25-year-old healthy male control (HC) were thawed and rested for 2 h at 37 °C and 5% CO_2_. Cells were then cultured in triplicates at 37 °C and 5% CO_2_ for 96 h in RPMI 1640 medium containing L-glutamine (supplemented with 10% FBS, penicillin/streptomycin, gentamicin, non-essential amino acids and sodium pyruvate) following polyclonal T cell activation using anti-CD3ε monoclonal antibody (OKT3, Invitrogen, 16–0037–81, 30 ng/ml). Cells were either cultured in the presence of sirolimus (Pfizer) at the noted concentrations or DMSO before flow cytometry.

### Analysis of cytokines/chemokines/soluble receptors

Cytokine, chemokine, and soluble receptor mediators were measured in cryopreserved plasma. All kit components from V-plex Ultra-Sensitive kit (Meso Scale Discovery, K15209D) were processed as per the manufacturer’s instructions. Electroluminescent data were analyzed with a four-parameter logistic curve fit using MSD Discovery Workbench.

### Statistical analysis

Data analysis and statistical methods were performed in GraphPad Prism 7 (La Jolla, CA). For multiple comparisons in the in vitro cell culture, one-way ANOVA was carried out, and post-hoc test *P* values are indicated for all comparisons reaching significance (α = 0.05).

### Reporting summary

Further information on research design is available in the Nature Research Reporting Summary linked to this article.

## Supplementary information

Supplementary Information

Reporting Summary

## Data Availability

The datasets generated during and analyzed during the current study are available from the corresponding author on reasonable request.
