# Supplementary material for: Targeting the mTOR pathway uncouples the efficacy and toxicity of PD-1 blockade in renal transplantation
Source: Nat Commun. 2019 Oct 17;10:4712. doi: 10.1038/s41467-019-12628-1 (PMC6797722; doi:10.1038/s41467-019-12628-1)
Supplement: Supplementary file 1 — Supplementary Information [file 41467_2019_12628_MOESM1_ESM.pdf]

Esfahani et al.

Targeting the mTOR pathway uncouples the efficacy and toxicity of PD-1 blockade in renal transplantation

SUPPLEMENTARY FILES

Supplementary Figure 1

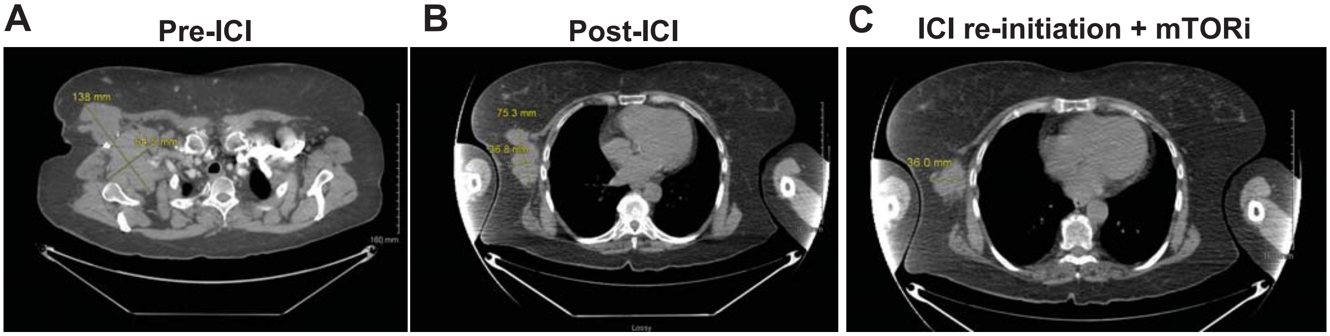

Supplementary Figure 1: RECIST imaging response of the patient's melanoma during her trajectory. (A) A large right-sided non-resectable axillary melanoma is noted. (B) A partial response of the tumor is noted at W7 of treatment, before the onset of irAEs. (C) Tumor control and ongoing allograft tolerance are maintained at W22 of treatment, following the resolution of irAEs and the introduction of ICI-mTORi combination therapy.

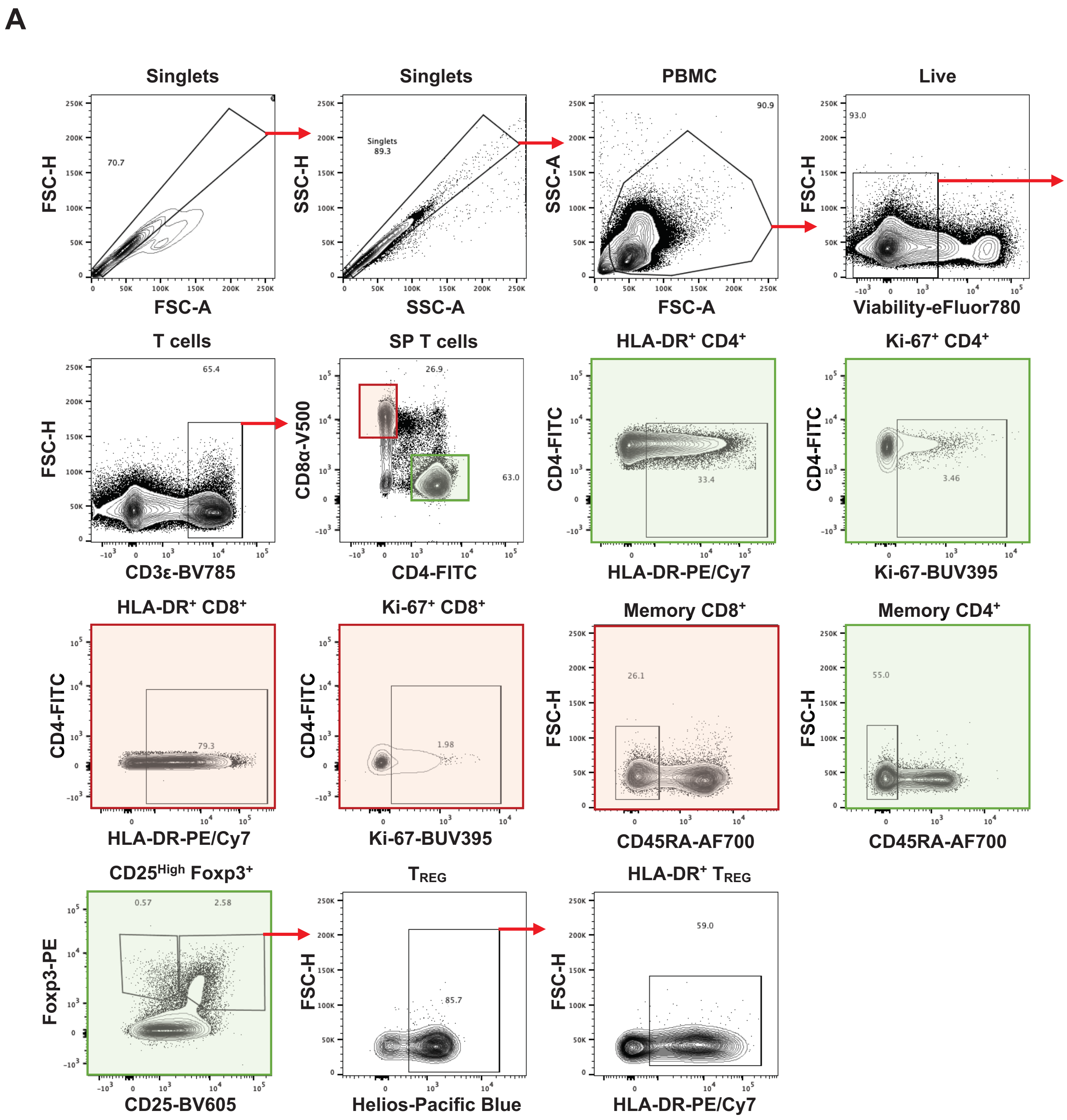

Supplementary Figure 2: Gating strategy for T cell activation panel. (A) PBMC from the patient were stained for ex vivo flow cytometric analysis of T cell activation. Live single cells were gated as CD3+ T cells and further subdivided into CD4 or CD8 single-positive (SP) T cells. On these SP T cell populations, activation was assessed by quantifying the proportion of cells expressing HLA-DR and Ki-67. Memory was assessed by CD45RA expression. From CD4+ T cells, activated TREG cells were defined as CD25High Foxp3 + Helios+ HLA-DR+ cells.

Supplementary Figure 3

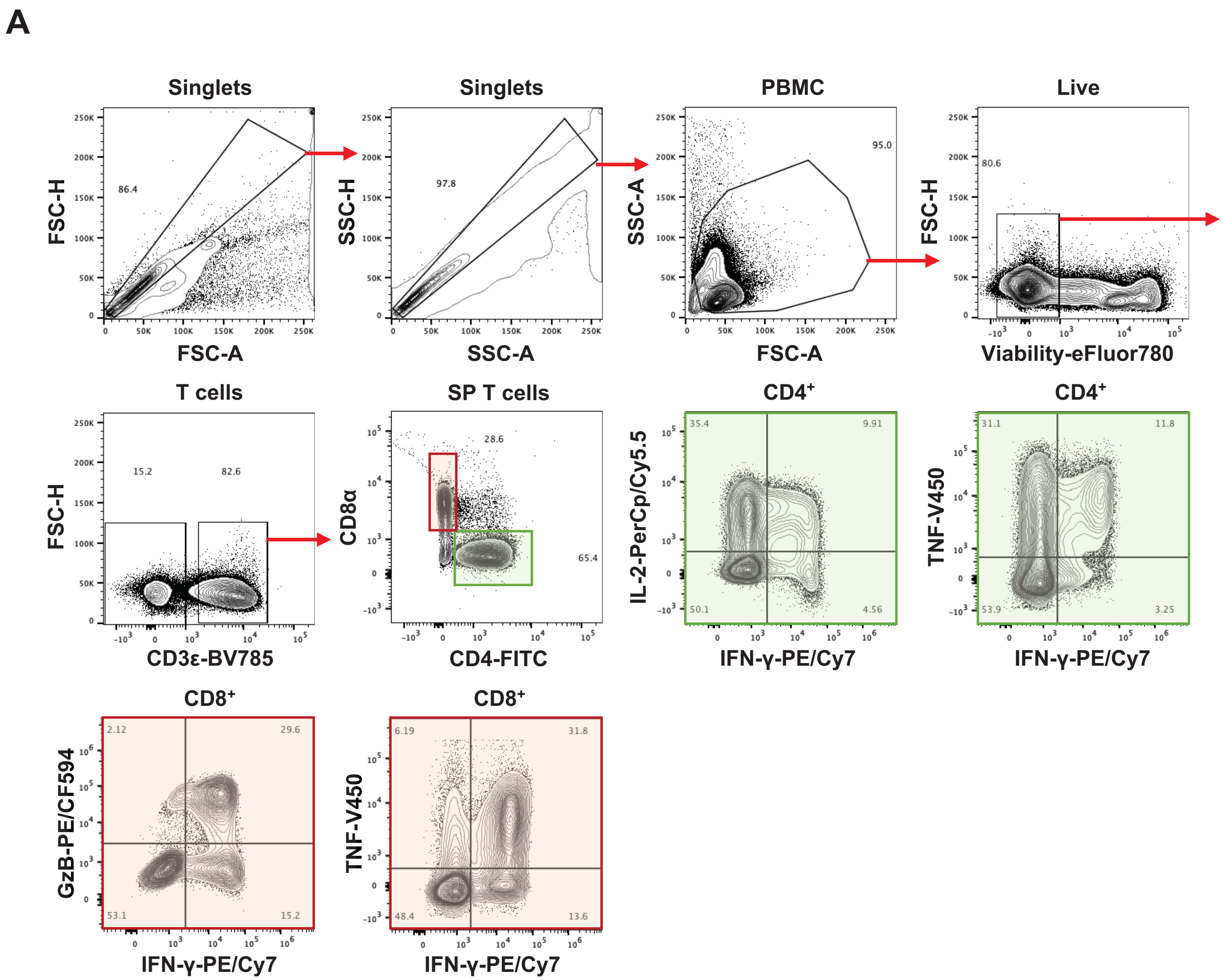

Supplementary Figure 3: Gating strategy for T cell cytokine secretion panel. (A) PBMC from the patient were stimulated with PMA, ionomycin and Golgi Stop for 4 hours before staining for flow cytometric analysis of cytokine expression. Live single cells were gated as CD3+ T cells and further subdivided into CD4 or CD8 SP T cells. From CD4+ T cells, proportions of cells expressing IL-2, IFN-γ and TNF were assessed. Proportions of CD8+ T cells expressing GzB, IFN-γ and TNF were quantified as well.

Supplementary Figure 4

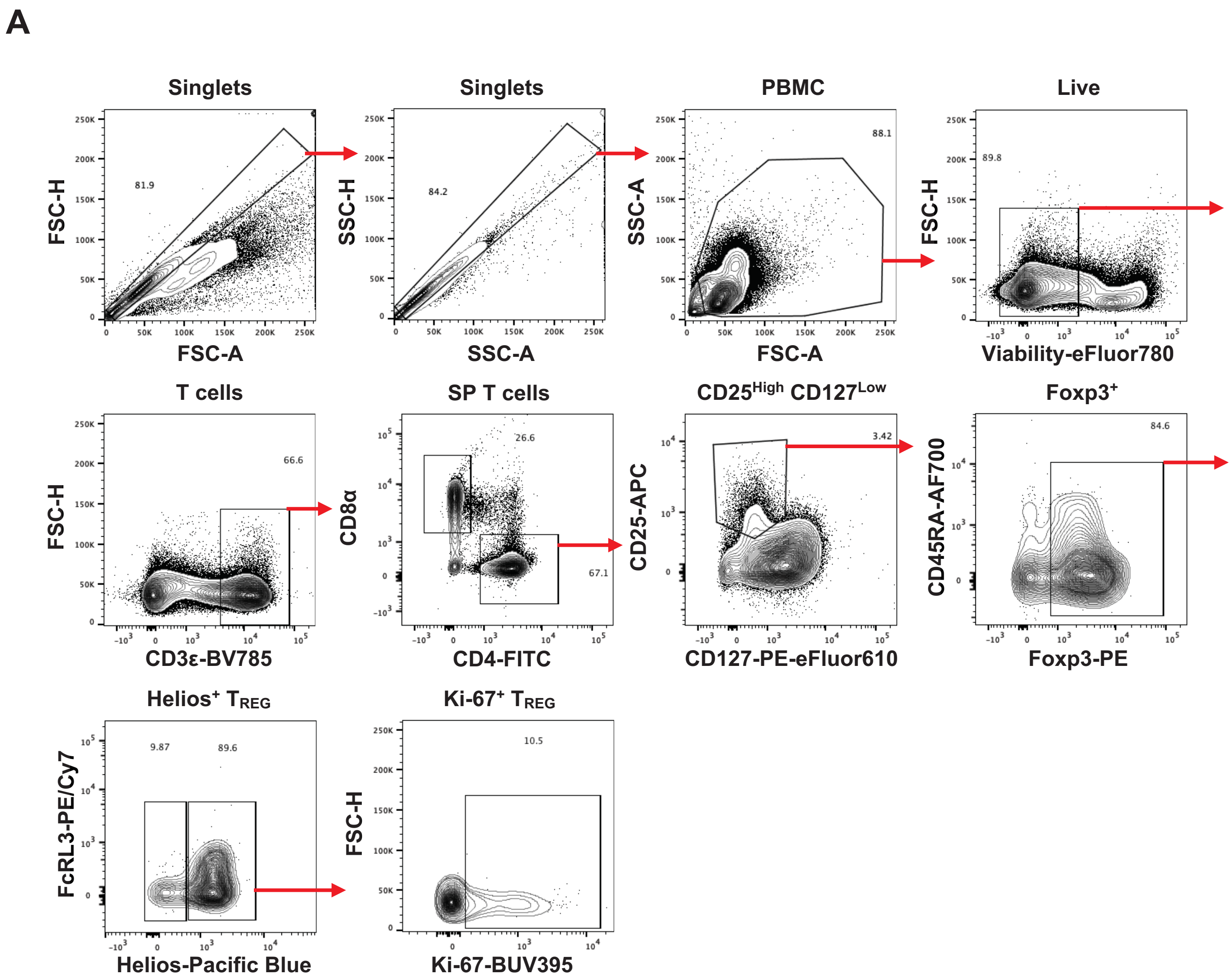

Supplementary Figure 4: Gating strategy for TREG cell panel. (A) PBMC from the patient were stained for ex vivo flow cytometric analysis of T cell activation. Live single cells were gated as CD3+ T cells and further subdivided into CD4 or CD8 single-positive (SP) T cells. From CD4+ T cells, TREG cells were defined as CD25High CD127Low Foxp3+ Helios+ cells.

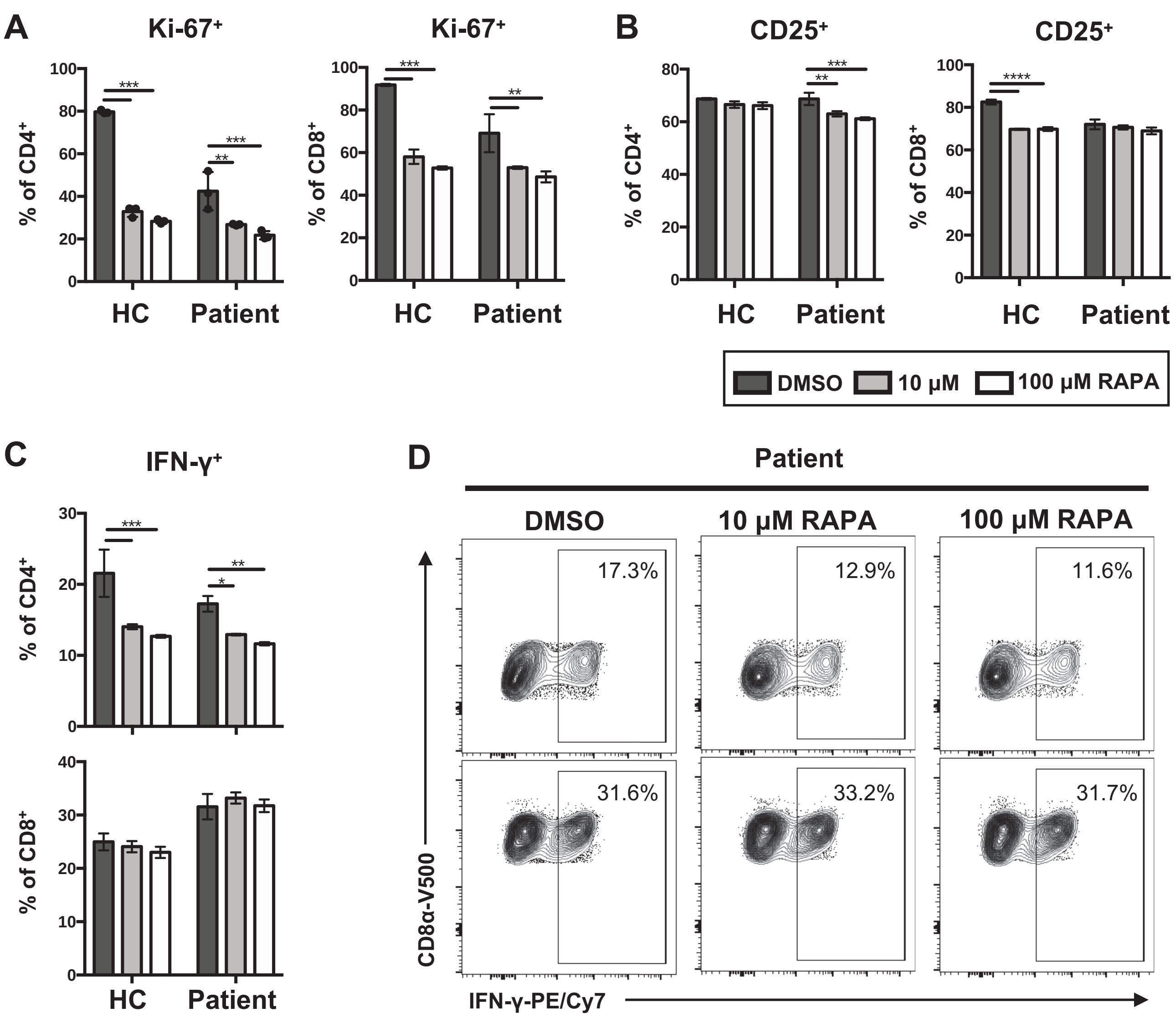

Supplementary Figure 5: In vitro use of sirolimus on patient PBMC decreases T cell activation but does not affect the frequency of IFN-g-producing T cells. T cells in patient PBMC and PBMC from a healthy control (HC) were polyclonally activated using anti-CD3ε monoclonal antibody (OKT3) and left in culture for 96 hours. Cells were subsequently stained for various T cell lineage-defining and activation markers. A subset of cells was also stimulated with PMA and ionomycin and treated with GolgiStop to assess cytokine production. (A) Proportions of cycling (Ki-67+) CD4+ and CD8+ T cells, (B) CD25+ (activated) CD4+ and CD8+ T cells, and (C) IFN-g+ CD4+ or CD8+ T cells are shown. (D) Flow cytometry plots for CD4+ (top three) and CD8+ (bottom three) T cell production of IFN-g are provided. The experiment was done in triplicates, mean ±SD are shown. P values were determined by Tukey's range test (*p<0.05, **p<0.01, ***p<0.001).
