# Supplementary material for: Targeting the mTOR pathway uncouples the efficacy and toxicity of PD-1 blockade in renal transplantation
Source: Nat Commun. 2019 Oct 17;10:4712. doi: 10.1038/s41467-019-12628-1 (PMC6797722; doi:10.1038/s41467-019-12628-1)
Supplement: Supplementary file 2 — Reporting Summary [file 41467_2019_12628_MOESM2_ESM.pdf]

nature research

Corresponding author(s): Ciriaco Piccirillo and Wilson Miller

Last updated by author(s): 7/8/2019

Reporting Summary

Nature Research wishes to improve the reproducibility of the work that we publish. This form provides structure for consistency and transparency in reporting. For further information on Nature Research policies, see Authors & Referees and the Editorial Policy Checklist.

Statistics

For all statistical analyses, confirm that the following items are present in the figure legend, table legend, main text, or Methods section.

n/a	Confirmed	
☐	☒	The exact sample size (n) for each experimental group/condition, given as a discrete number and unit of measurement
☐	☒	A statement on whether measurements were taken from distinct samples or whether the same sample was measured repeatedly
☐	☒	The statistical test(s) used AND whether they are one- or two-sided<br>*Only common tests should be described solely by name; describe more complex techniques in the Methods section.*
☐	☒	A description of all covariates tested
☒	☐	A description of any assumptions or corrections, such as tests of normality and adjustment for multiple comparisons
☐	☒	A full description of the statistical parameters including central tendency (e.g. means) or other basic estimates (e.g. regression coefficient) AND variation (e.g. standard deviation) or associated estimates of uncertainty (e.g. confidence intervals)
☐	☒	For null hypothesis testing, the test statistic (e.g. F, t, r) with confidence intervals, effect sizes, degrees of freedom and P value noted<br>*Give P values as exact values whenever suitable.*
☒	☐	For Bayesian analysis, information on the choice of priors and Markov chain Monte Carlo settings
☒	☐	For hierarchical and complex designs, identification of the appropriate level for tests and full reporting of outcomes
☒	☐	Estimates of effect sizes (e.g. Cohen's d, Pearson's r), indicating how they were calculated

Our web collection on statistics for biologists contains articles on many of the points above.

Software and code

Policy information about availability of computer code

Data collection	Flow cytometry data was collected on a BD LSRFortessa X-20 using the BD FACSDiva software (BD Biosciences).
Data analysis	Flow cytometry data was analyzed on FlowJo v.10 (FlowJo, LLC). Statistical tests for flow cytometric data were analyzed on Prism 7 (GraphPad).

For manuscripts utilizing custom algorithms or software that are central to the research but not yet described in published literature, software must be made available to editors/reviewers. We strongly encourage code deposition in a community repository (e.g. GitHub). See the Nature Research guidelines for submitting code & software for further information.

Data

Policy information about availability of data

All manuscripts must include a data availability statement. This statement should provide the following information, where applicable:

- Accession codes, unique identifiers, or web links for publicly available datasets
- A list of figures that have associated raw data
- A description of any restrictions on data availability

Provide your data availability statement here.

Field-specific reporting

Please select the one below that is the best fit for your research. If you are not sure, read the appropriate sections before making your selection.

☒ Life sciences ☐ Behavioural & social sciences ☐ Ecological, evolutionary & environmental sciences

For a reference copy of the document with all sections, see nature.com/documents/nr-reporting-summary-flat.pdf

Life sciences study design

All studies must disclose on these points even when the disclosure is negative.

Sample size	The submitted manuscript is a report of a unique case wherein immune checkpoint inhibition for the treatment of melanoma resulted in substantial immune-related adverse events. Given the rarity of such a case and the descriptive nature of our report, a sample size of 1 is given.
Data exclusions	No data were excluded.
Replication	For in vitro experiments and the v-plex meso scale discovery immunoassay, samples were tested in duplicates. For immunophenotyping of patient PBMC, only certain markers (e.g. CD3, CD4, CD8, Ki-67) could be tested in duplicates due to repetitions of these markers in multiple panels. If replicates were available they were shown as such in the figures.
Randomization	Not applicable. The submitted manuscript is a detailed description of a single patients' peripheral immune phenotype during a treatment course for melanoma and autoimmunity.
Blinding	Blinding was not applicable for the immunophenotyping of patient PBMC and the v-plex meso scale discovery as there was only a single individual being looked at.

Reporting for specific materials, systems and methods

We require information from authors about some types of materials, experimental systems and methods used in many studies. Here, indicate whether each material, system or method listed is relevant to your study. If you are not sure if a list item applies to your research, read the appropriate section before selecting a response.

Materials & experimental systems

n/a	Involved in the study	
☐	☒	Antibodies
☒	☐	Eukaryotic cell lines
☒	☐	Palaeontology
☒	☐	Animals and other organisms
☐	☒	Human research participants
☐	☒	Clinical data

Methods

n/a	Involved in the study	
☒	☐	ChIP-seq
☐	☒	Flow cytometry
☒	☐	MRI-based neuroimaging

Antibodies

Antibodies used	Anti-CD3 BV785 (Biolegend, cat: 317330, OKT3, lot: B255877), anti-CD4 FITC (BD Biosciences, cat: 561842, RPA-T4, lot: 5097644), anti-CD8a V500 (BD Biosciences, cat: 560774, RPA-T8, lot: 7158969), anti-CD25 APC (BD Biosciences, cat: 555434, M-A251, lot: 8073639), anti-CD25 BV605 (BD Biosciences, cat: 562660, 2A3, lot: B256321), anti-CD127 PE-eFluor610 (Invitrogen, cat: 61-1278-42, eBioRDR5, lot: 4324664), anti-CD45RA Alexa Fluor 700 (BD Biosciences, cat: 560673, HI100, lot: 7180940), anti-TIGIT PerCp-eFluor710 (Invitrogen, cat: 46-9500-42, MBSA43, lot: 4318928), anti-CD307c biotinylated (BD Biosciences, cat: 565056, H5, lot: 7145642), anti-HLA-DR biotinylated (Invitrogen, cat: 13-9956-82, LN3, lot: 4300259), anti-Foxp3 PE (Invitrogen, cat: 12-4777-42, 236A/E7, lot: 1996386), anti-Helios PacificBlue (Biolegend, cat: 137220, 22F6, lot: B238304), anti-Ki-67 BUV 395 (BD Biosciences, cat: 564071, B56, lot: 8145992), anti-IL-2 PerCP/Cy5.5 (BD Biosciences, cat: 560708, MQ1-17H12, lot: 8142599), anti-IFN-gamma PE/Cy7 (BD Biosciences, cat: 560741, 4S.B3, lot: 7258713), anti-TNF V450 (BD Biosciences, cat: 561311, MAb11, lot: 8018611), anti-Granzyme B PE-CF594 (BD Biosciences, cat: 562462, GB11, lot: 7215784).
Validation	All antibodies were validated by the manufacturer for detection of human proteins and use in flow cytometry. All the antibodies are regularly used by our group and have been used in numerous publications in the past.

Human research participants

Policy information about studies involving human research participants

Population characteristics	The healthy control analyzed in supplemental figure 1 was a 26-year-old male with no known genotypic abnormalities and a healthy medical history.
Recruitment	*Describe how participants were recruited. Outline any potential self-selection bias or other biases that may be present and how these are likely to impact results.*
Ethics oversight	*Identify the organization(s) that approved the study protocol.*

Note that full information on the approval of the study protocol must also be provided in the manuscript.

Clinical data

Policy information about clinical studies

All manuscripts should comply with the ICMJE guidelines for publication of clinical research and a completed CONSORT checklist must be included with all submissions.

Clinical trial registration	N/A
Study protocol	This is a case study
Data collection	Data collection started after signature of informed consent, and continued through the patient's clinical trajectory
Outcomes	For this case study, the two outcomes under assessment were allograft tolerance and tumor response.

Flow Cytometry

Plots

Confirm that:

☒ The axis labels state the marker and fluorochrome used (e.g. CD4-FITC).

☒ The axis scales are clearly visible. Include numbers along axes only for bottom left plot of group (a 'group' is an analysis of identical markers).

☒ All plots are contour plots with outliers or pseudocolor plots.

☒ A numerical value for number of cells or percentage (with statistics) is provided.

Methodology

Sample preparation	PBMC were isolated from patient blood using Ficoll-paque centrifugation. Samples were then frozen and then thawed when needed for flow cytometry. Before flow cytometric staining, cells were rested for 2 hours at 37 degrees Celsius and 5% CO2. In order to visualize cytokine production by flow cytometry, some cells were further stimulated with PMA, ionomycin and GolgiStop (BD Biosciences) for 4 hours before flow cytometry staining.
Instrument	BD LSRFortessa X-20
Software	Flow cytometry data was collected by BD FACSDiva software and analyzed by FlowJo v.10.
Cell population abundance	Cells were not sorted.
Gating strategy	Please see supplemental information for the gating strategies.

☒ Tick this box to confirm that a figure exemplifying the gating strategy is provided in the Supplementary Information.
